# Improved assessment of aortic 3D blood flow with combined k-t accelerated 3D CINE bSSFP & 4D flow MRI

**DOI:** 10.1186/1532-429X-18-S1-P232

**Published:** 2016-01-27

**Authors:** Kelly B Jarvis, Can Wu, Shivraman Giri, Susanne Schnell, Alex J Barker, Jeremy D Collins, James C Carr, Michael Markl

**Affiliations:** 1 Department of Radiology, Northwestern University, Chicago, IL USA; 2 Siemens Healthcare, Chicago, IL USA; 3 Department of Biomedical Engineering, Northwestern University, Chicago, IL USA

## Background

To determine hemodynamic parameters from 4D flow MRI, vessel boundaries are typically depicted by calculating a 3D phase contrast MR angiogram (PC-MRA) from 4D flow magnitude and flow images. However, this approach is limited by 1) low blood-tissue contrast of the magnitude images and 2) velocity weighting of PC-MRA may not fully depict areas of slow or swirling (vortex) flow. As a result, 3D flow analysis at the aortic root has been challenging due to the presence of vortex flow distal to the aortic valve. Fast 3D balanced steady state free precession (bSSFP) time-resolved MRI sequences offer a promising alternative, providing anatomical images with improved blood-tissue contrast. The goal of this feasibility study was to integrate results from 3D CINE bSSFP with 4D flow MRI to improve the segmentation of vessel anatomy and thus 3D blood flow assessment compared to standard segmentation using PC-MRA data alone.

## Methods

Non-contrast free-breathing 3D CINE bSSFP and 4D flow MRI were acquired in the aorta (n = 6 controls: age=56+/-11 yrs, 1 female) with identical spatial resolution (Figure 1) and k-t acceleration with reduction factor R=5 to reduce overall scan times. Multiple 3D CINE bSSFP scans were averaged to mitigate breathing effects and then interpolated along the temporal domain for integration with 4D flow. 3D segmentation of the aorta included 1) standard approach using the PC-MRA and 2) new approach utilizing 3D CINE bSSFP MRI averaged over the cardiac cycle. The resulting aortic segmentations were used to mask the 4D flow velocity data for analysis. Systolic blood flow was visualized using streamlines emitted from the aortic volume and graded for the appearance of vortex flow in the sinuses of Valsalva (0=none, 1=mild, 2=moderate). Flow and peak velocity were quantified in the sinuses and compared between methods using the paired Wilcoxon signed rank test.

**Figure:**
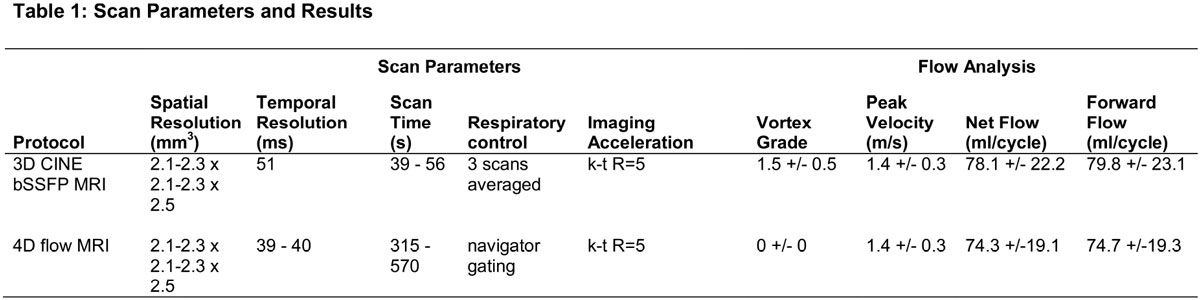
Figure 1

## Results

**Figure 2:**
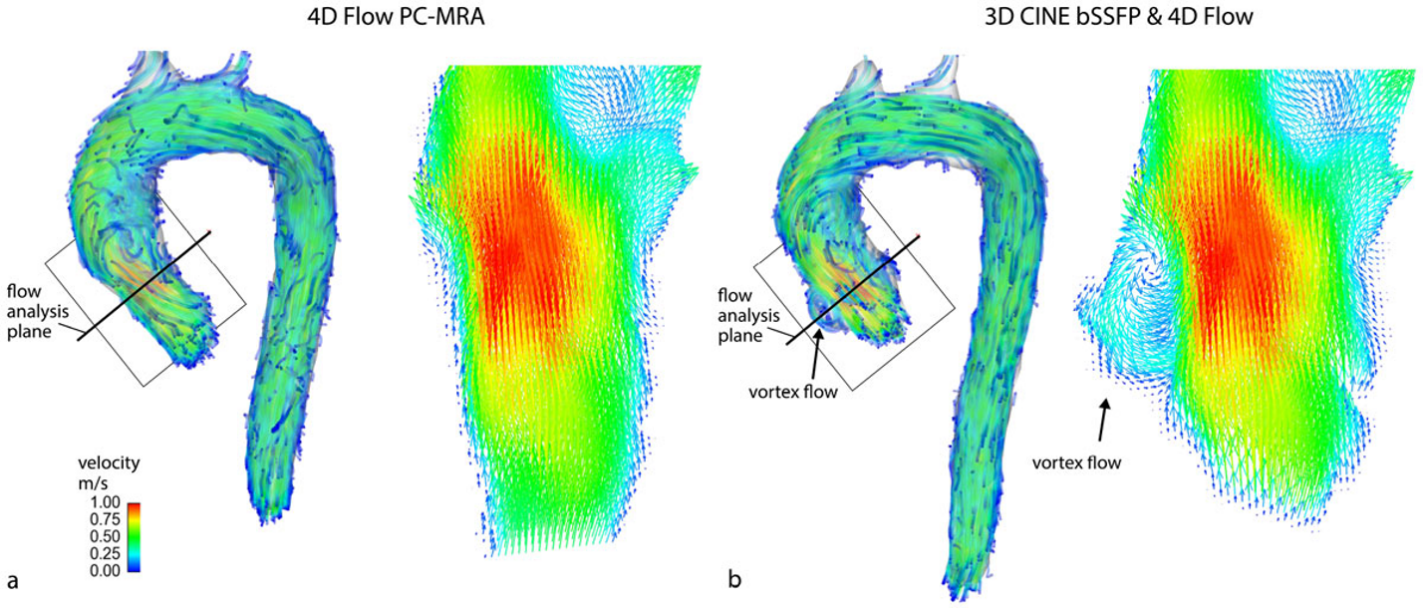
**3D CINE bSSFP enhanced flow visualization**. Flow was visualized at one systolic timepoint for an aortic volume segmented from a) the 4D flow PC-MRA and b) time-averaged 3D CINE bSSFP MRI. On the left: streamlines were emitted from the aortic volume (shown in light gray) and color-coded by velocity. The flow analysis planes shown are the locations where flow quantifications were made. On the right: to further illustrate the magnitude and direction of flow, velocity arrows are shown at a cut-plane perpindicular to the analysis plane. With 3D CINE bSSFP MRI the segmentation is enhanced in the aortic root, enabling the visualization of vortex flow in the sinuses of Valsalva (black arrows).

3D CINE bSSFP MRI enabled the segmentation of the aorta, including areas of vortex flow distal to the aortic valve in the sinuses of Valsalva (figure 2). With the standard segmentation approach using the PC-MRA, vortex flow in the sinuses could not be reliably visualized for any subject. In contrast, the new approach utilizing combined 3D CINE bSSFP and 4D flow data enabled the visualization of either mild or moderate vortex flow in the sinuses for all subjects. As expected, peak velocity remained the same between segmentation techniques. Also, flow results were comparable (p>0.05) (Figure 1).

## Conclusions

We have developed k-t accelerated 3D CINE bSSFP MRI compatible for post-processing combination with 4D flow MRI. This technique shows great potential to improve the hemodynamic assessment of patients with aortic disease by providing an accurate segmentation of vessel walls in areas such as the aortic root that are difficult to assess using standard methods. Further study is needed to optimize the scan protocol, test alternative PC-MRA calculations and explore time-resolved segmentation using 3D CINE bSSFP MRI.

